# Outcomes of a Presurgical Optimization Program for Elective Hernia Repairs Among High-risk Patients

**DOI:** 10.1001/jamanetworkopen.2021.30016

**Published:** 2021-11-01

**Authors:** Lia D. Delaney, Ryan Howard, Krisinda Palazzolo, Anne P. Ehlers, Shawna Smith, Michael Englesbe, Justin B. Dimick, Dana A. Telem

**Affiliations:** 1 Medical School, University of Michigan, Ann Arbor; 2 Center for Healthcare Outcomes and Policy, University of Michigan, Ann Arbor; 3 Division of Minimally Invasive Surgery, Department of Surgery, University of Michigan, Ann Arbor; 4 Department of Health Management and Policy, School of Public Health, University of Michigan, Ann Arbor

## Abstract

**Importance:**

Preoperative optimization is an important clinical strategy for reducing morbidity; however, nearly 25% of persons undergoing elective abdominal hernia repairs are not optimized with respect to weight or substance use. Although the preoperative period represents a unique opportunity to motivate patient health behavior changes, fear of emergent presentation and financial concerns are often cited as clinician barriers to optimization.

**Objective:**

To evaluate the feasibility of evidence-based patient optimization before surgery by implementing a low-cost preoperative optimization clinic.

**Design, Setting, and Participants:**

This quality improvement study was conducted 1 year after a preoperative optimization clinic was implemented for high-risk patients seeking elective hernia repair. The median (range) follow-up was 197 (39-378) days. A weekly preoperative optimization clinic was implemented in 2019 at a single academic center. Referral occurred for persons seeking elective hernia repair with a body mass index greater than or equal to 40, age 75 years or older, or active tobacco use. Data analysis was performed from February to July 2020.

**Exposures:**

Enrolled patients were provided health resources and longitudinal multidisciplinary care.

**Main Outcomes and Measures:**

The primary outcomes were safety and eligibility for surgery after participating in the optimization clinic. The hypothesis was that the optimization clinic could preoperatively mitigate patient risk factors, without increasing patient risk. Safety was defined as the occurrence of complications during participation in the optimization clinic. The secondary outcome metric centered on the financial impact of implementing the preoperative optimization program.

**Results:**

Of the 165 patients enrolled in the optimization clinic, most were women (90 patients [54.5%]) and White (145 patients [87.9%]). The mean (SD) age was 59.4 (15.8) years. Patients’ eligibility for the clinic was distributed across high-risk criteria: 37.0% (61 patients) for weight, 26.1% (43 patients) for tobacco use, and 23.6% (39 patients) for age. Overall, 9.1% of persons (15 patients) were successfully optimized for surgery, and tobacco cessation was achieved in 13.8% of smokers (8 patients). The rate of hernia incarceration requiring emergent surgery was 3.0% (5 patients). Economic evaluation found increased operative yield from surgical clinics, with a 58% increase in hernia-attributed relative value units without altering surgeon workflow.

**Conclusions and Relevance:**

In this quality improvement study, a hernia optimization clinic safely improved management of high-risk patients and increased operative yield for the institution. This represents an opportunity to create sustainable and scalable models that provide longitudinal care and optimize patients to improve outcomes of hernia repair.

## Key Points

**Question:**

Can a low-cost optimization clinic be implemented to successfully manage high-risk patients seeking hernia repair?

**Findings:**

In this quality improvement study, 1 year after the implementation of a preoperative optimization clinic, 9.1% of high-risk patients became eligible for hernia repair through the optimization of a high-risk characteristic, and the number of emergency surgery events was low.

**Meaning:**

These findings suggest that this model represents a scalable and sustainable framework for preoperative optimization with the potential to improve the care of patients with hernias.

## Teaser

This quality improvement study evaluates the feasibility of a low-cost preoperative optimization clinic that was implemented to improve the care of high-risk patients seeking elective hernia repair.

## Introduction

Ventral and incisional hernia repair is one of the most common operations performed annually in the US, with an estimated 350 000 procedures performed each year.^1^ Despite their ubiquity and ability to improve quality of life, they are associated with a high incidence of postoperative complications.^2,3^ Efforts to mitigate the risk of these postoperative outcomes traditionally have focused on perioperative processes and postoperative rescue. Much less attention has been given to the critical decision of when and on whom to operate, which is inextricably linked to patient outcomes. It is well accepted that addressing modifiable factors, such as substance abuse, glycemic control, and obesity, can improve outcomes such as length of stay and return to baseline functional status and decrease health care costs.^4,5,6^ In addition, the preoperative period has been identified as a unique opportunity to motivate patient health behavior changes.^7,8,9^ Despite this, there is a pervasive lack of adherence to evidence-based practices in decisions to delay surgery until risk factors are appropriately mitigated, and nearly 25% of persons undergoing elective abdominal hernia repairs are not optimized with respect to weight or substance use.^10,11,12,13^

Although improving and even standardizing preoperative care and decision-making in this area may represent an effective avenue by which to improve overall surgical quality, there are only limited data as to the results of this type of intervention. Simply denying operative management for high-risk patients, while avoiding the risks associated with surgery, is associated with poor quality of life.^14^ Moreover, the safety of delaying operations with regard to emergent surgery and/or visits related to incarceration and other hernia-related complications remains largely unknown. Although a handful of small-scale, single-institution initiatives demonstrated that weight reduction was associated with modest improvements in 30-day postoperative hernia outcomes,^15^ the effectiveness and benefits of other interventions targeted at high-risk patients before hernia repair, such as preoperative smoking cessation and even physical activity, are less well-described. The cost of these types of interventions is also unknown. In addition, previous data demonstrated that financial concerns may play a role in surgeon decision-making for abdominal wall hernia repair.^3^

In this context, we designed and implemented a hernia optimization program with the intent of providing a surgical home with longitudinal follow-up for persons who would benefit from optimization before surgery. This program expanded traditional preoperative patient care and provided an opportunity to explore the effectiveness and safety of optimization and delayed surgery in a high-risk patient population. Moreover, we sought to evaluate the institutional costs and economic impact of a hernia preoperative optimization program.

## Methods

### Patient Cohort and Optimization Clinic

The primary intervention was the implementation of a preoperative optimization clinic at the University of Michigan, a large, academic, tertiary care hospital and high-volume surgical center. The clinic consisted of a half-day clinic every week staffed by a physician’s assistant as the primary clinician to address comorbidities in a longitudinal manner. All patients referred to our hernia clinic were automatically screened via telephone triage for active tobacco use, morbid obesity, or advanced age on the basis of their medical record. This study received exemption status from the University of Michigan institutional review board for being secondary research for which patient consent was not required given that the research used previously collected health information for research purposes only. This study followed the Standards for Quality Improvement Reporting Excellence (SQUIRE) reporting guideline.^16^

A telephone decision tree was designed and implemented into the hospital’s surgical call center in December 2018 to screen high-risk patients seeking abdominal wall hernia repair. Persons with groin hernias were excluded from the decision tree. Patient eligibility was based on the presence of at least 1 of the following characteristics: body mass index (BMI; calculated as weight in kilograms divided by height in meters squared) greater than or equal to 40, age greater than or equal to 75 years, or active tobacco use (which was defined as ongoing smoking, smokeless tobacco use, or a positive urine cotinine test). Although age is a nonmodifiable risk factor, in this case it served as a proxy for identifying patients with a higher likelihood of overall frailty or physical deconditioning, which is modifiable in the preoperative period.^4,17^ Once scheduled for an appointment, the advanced practice clinician was able to fully address a patient’s functional capacity and frailty, as well as complete a thorough risk assessment based on medical history, and subsequently triage them to appropriate resources if there were modifiable high-risk characteristics that colocated with age. Race and ethnicity were included in this study in order to best understand the patient population engaged by this clinic, and demographics were identified by patient medical records.

If a patient screened positive for one of these risk factors, instead of having an appointment scheduled with a surgeon, they were given the option to schedule a 30-minute appointment in our optimization clinic (Figure). At the first appointment, their risk factors were further characterized, and an individualized optimization goal was agreed upon between the clinician and patient. For example, if a patient was an active tobacco user, their readiness to quit was evaluated and they received appropriate resources including motivational cessation counseling, a referral to an institutional Quit Tobacco Program, or a prescription for a pharmacological intervention such as varenicline or nicotine patches. If a patient was referred for obesity, they received weight loss counseling, including nutrition and exercise guidance, as well as specialist referrals to a dietician or bariatric surgeon if desired. If a patient was referred for being aged 75 years or older, their baseline functional status was assessed, and they were screened for frailty during the appointment. Follow-up was conducted via in-person appointments and telephone calls every 6 to 8 weeks. At each follow-up encounter, a patient’s progress was evaluated, and they were provided with additional recommendations. If a patient was determined to have met their predetermined health goal at any time, they were re-referred for surgical evaluation. If patients made the decision to no longer pursue surgery, they were provided any further resources or referrals that might help them achieve their health goals and instructed to reengage with the program at any time. There was no minimum or limit on the number of visits that patients could attend, and during the pilot period, patients were encouraged to continue to stay engaged in the program and optimization efforts, regardless of the time enrolled. The cadence of in-person visits was patient dependent and based on their preference, to represent pragmatic real-world conditions.

**Figure.**
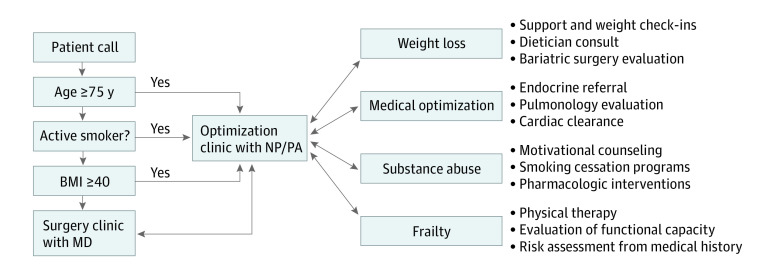
Optimization Clinic Decision Tree BMI indicates body mass index (calculated as weight in kilograms divided by height in meters squared); MD, medical doctor; NP, nurse practitioner; PA, physician’s assistant.

The multidisciplinary resources of a large academic institution were made available to patients at these appointments. The clinic allowed the potential for coordination with a given patient’s other clinicians for an interdisciplinary management of comorbidities. The clinic was also an opportunity to discuss participants’ health and quality-of-life goals while providing education on nonoperative management options for asymptomatic patients. Before implementation of the optimization clinic, all patients seeking elective hernia repair were seen by a surgeon in the surgical clinic, and the presence of high-risk comorbidities and subsequent eligibility for surgery were determined during the clinic appointment.

One year after clinic implementation, patient health outcomes were evaluated. Patient data were abstracted from electronic medical records. Patients were included in the analysis if they attended and completed at least 1 clinic visit in the hernia optimization program at the University of Michigan from January 1, 2019, to December 31, 2019. Any cancelled or no-show appointments were excluded. A retrospective medical record review was conducted to determine the presenting patient comorbidity (BMI, tobacco use, or age) for program participation. All patient encounters after the initial clinic visit were reviewed to obtain individual health outcome data. Patients who presented to the clinic and were determined not to have clinical evidence of a hernia through imaging and physical examination were not followed up in the program or included in health outcome analyses.

### Outcomes

Our primary outcomes were safety and subsequent eligibility for surgery after participating in the optimization clinic. We hypothesized that the optimization clinic could preoperatively mitigate patient risk factors without increasing patient risk. Specifically, we examined smoking cessation or weight loss that resulted in a change in patient surgical eligibility with or without the occurrence of a completed surgery at the University of Michigan or an outside hospital. Safety was defined as the occurrence of complications during participation in the optimization clinic, including hernia-related emergency department visit, all-cause emergency department visit, hospital admissions, hernia incarceration events, and emergency hernia surgery. The Care Everywhere feature of the electronic medical record allowed querying of patient data at other institutions and was used to capture all patient interactions with other hospitals in the state of Michigan.

Our secondary outcome metric centered on the financial impact of implementing the preoptimization program. We hypothesized that triaging high-risk patients for preoperative optimization not only would benefit the patient but also create additional capacity in the surgical clinic and increase operative yield. To evaluate this hypothesis, we calculated the relative value units (RVUs) for new patient hernia clinic visits booked into open spaces during the year 2019 that resulted in a completed hernia operation in 2019. As a comparison, generated RVUs were also calculated for surgical clinic visits that resulted in a hernia operation in 2018, before the implementation of the optimization clinic. Surgical yield was attributed to 2018 as long as patients were seen in clinic in 2018. Similarly, RVUs generated from surgical cases in 2019 were attributed to the 2019 cohort if the patient had initiated care in the year 2019. RVUs generated from the optimization clinic were not included in the 2019 RVU total. The year 2018 was chosen as the sole comparison because the surgical division was consistent in both surgeon number and operating room time from 2018 to 2019. There were no other competing efforts or interventions in the minimally invasive surgery division in either 2018 or 2019. Any incremental change in clinic capacity and RVUs from 2018 to 2019 was analyzed.

### Statistical Analysis

All statistical analyses were descriptive and were performed using Stata statistical software version 13.1 (StataCorp). Data analysis was performed from February to July 2020.

## Results

In 2019, 176 patients were referred to and attended the hernia optimization clinic. Ten patients did not have clinical evidence of a hernia and did not participate further in the program, and 1 patient left the clinic before being seen, leaving 165 participants included in the analysis (90 women [54.5%]; mean [SD] age, 59.4 [15.8] years; mean [SD] BMI, was 37.6 [10.5]). The majority of the patients were White (145 patients [87.9%]). Full demographic characteristics of the cohort are listed in Table 1.

**Table 1.** Demographic Characteristics of Cohort

Characteristic	Patients, No. (%) (N = 165)
Age, y	
<50	46 (27.9)
50-74	76 (46.1)
≥75	43 (26.1)
Sex	
Male	75 (45.5)
Female	90 (54.5)
Race	
Black	14 (8.5)
White	145 (87.9)
Other^a^	6 (3.6)
Body mass index^b^	
<25	22 (13.3)
25-34.9	47 (28.5)
35-39.9	22 (13.3)
≥40	74 (44.6)
Active tobacco use	56 (33.9)

^a^ Other includes patients who identified as Chaldean, Hispanic, any other race or ethnicity, or did not answer.

^b^ Body mass index is calculated as weight in kilograms divided by height in meters squared.

Enrollment criteria for the optimization clinic included 61 patients (37.0%) with a BMI of 40 or higher, 43 patients (26.1%) with active tobacco use, and 39 patients (23.6%) aged 75 years or older (Table 2). Sixteen patients had more than 1 risk factor of eligibility for the program. In the absence of these 3 specific criteria, an additional 6 patients (3.6%) underwent physician-directed referral based on the presence of a medical history and/or multiple comorbidities that were judged to be sufficiently complicated to potentially affect surgical repair. The median (range) follow-up was 197 (39-378) days. Of the 165 patients evaluated for hernia repair, 115 (69.7%) had ventral or incisional hernias, and 44 (26.7%) had umbilical hernias. Although the telephone triage was meant to exclude groin hernias, 6 patients (3.6%) had femoral or inguinal hernias but remained enrolled in the optimization clinic and were included in the final analysis.

**Table 2.** Participant Enrollment Characteristics

Primary eligibility criteria	Patients, No. (%) (N = 165)
Body mass index ≥40^a^	61 (37.0)
Age ≥75 y	39 (23.6)
Tobacco use	43 (26.1)
Medical history	6 (3.6)
>1 High-risk characteristic	16 (9.7)
Hernia type	
Ventral or incisional	115 (69.7)
Umbilical	44 (26.7)
Inguinal	5 (3.0)
Femoral	1 (0.6)

^a^ Body mass index is calculated as weight in kilograms divided by height in meters squared.

As of February 1, 2020, 15 patients (9.1%) participating in the program successfully became eligible for surgery. Twelve of these patients, 7.3% of the cohort, qualified through direct mitigation or optimization of a high-risk characteristic that prompted their enrollment in the clinic. The plurality of these patients became optimized through tobacco cessation, as determined by a negative urine cotinine test, which was achieved by 8 (13.8%) participating active smokers. Three patients (4.7%) achieved eligibility through weight loss, 2 of whom were referred for a BMI of 40 or higher and 1 who was originally referred for a complicated medical history. Eligibility through weight loss was decided at the surgeon’s discretion once a patient’s BMI was less than 40. In addition, 8 participants (10.8%) with comorbid obesity actively pursued bariatric surgery upon referral. Three patients (7.7%) referred to the clinic for being older than 75 years were determined to be eligible for surgery after the follow-up and thorough evaluation of their functional status that the preoperative clinic allowed. Five patients (3.0%) received second surgical opinions at an outside institution. One patient (20.0%) underwent surgery at an outside institution and was not considered optimized in this analysis. The 4 other patients (80.0%) continued care through the optimization program. Table 3 stratifies optimization outcomes by presenting risk factor.

**Table 3.** Optimization Outcome Stratified by Risk Factor

Risk factor	Patients, No.	Patients, No. (%)	
		Qualified for surgery	Enrollment risk factor optimized
All patients^a^	165	15 (9.1)	12 (7.3)
Body mass index	63	3 (4.7)	3 (4.7)
Age^b^	39	3 (7.6)	0
Tobacco use	58	8 (13.8)	8 (13.8)
Medical history^b^	5	1 (20.0)	0

^a^ Of 165 patients who were enrolled in the program, 15 (9.1%) had become eligible for surgery at the time of follow-up. Twelve of these patients, 7.3% of the cohort, qualified through mitigation or optimization of the high-risk characteristic that was their eligibility criteria for the clinic.

^b^ Although age and medical history are not modifiable risk factors, 2 patients who were enrolled for being aged 75 years and older and 1 patient who was enrolled for a complicated medical history underwent frailty evaluation and became sufficiently physically conditioned for surgery.

At the time of follow-up, the incidence of emergent surgery was 3.0% (5 patients). All hospital presentations during clinic enrollment are outlined in the eTable in the Supplement. During the follow-up period, 11 patient presentations to the emergency department occurred because of patient concern for a hernia-related complication. Six of these patient presentations (54.5%) were determined to not be associated with their hernia. Five patients (45.5%) experienced hernia incarceration events requiring emergency repair, 2 of which (40.0%) were performed at outside institutions. Two of the 5 patients who underwent emergency surgery (40.0%) had postoperative complications, including both an emergency department presentation and a hospital admission within 30 days. Two patients (1.2%) were deceased at the time of follow-up, from health complications unrelated to their hernia.

Economic evaluation revealed increased surgical yield at the hospital’s main hernia clinic. The optimization clinic saw 176 patients who would have otherwise been seen in a surgical hernia clinic, which allowed more appointment times to be opened and designated for new, expedited patients with hernias. The 2019 University of Michigan minimally invasive surgical division saw 11% more new expedited patients with hernias overall (214 vs 193 patients) and had a 19% increase in operative yield. The incremental increase in hernia-attributed RVUs was 58% (from 980 in 2018 to 1545 in 2019) (Table 4). The cost of clinic operations included 0.1 full-time equivalent of the advanced practice clinician salary and benefits, which was approximately $10 000 in personnel cost. Cost per patient, including salaries, supplies, and clinic space, was calculated to be $236.00. Overall, clinic operations for 1 calendar year required an investment of approximately $41 536 and had a 58% increase in generated RVUs, compared with the previous year. During this time, there were no changes to the number of operating surgeons and their contribution the division.

**Table 4.** RVUs Generated From New Patients With Hernias Before (2018) and After (2019) Implementation of Optimization Clinic

Variable	RVUs, No.		Increase, %
	2018	2019	
Clinic appointments	193	214	11
Operations performed	75	89	19
Generated RVUs	980	1545	58

Abbreviation: RVU, relative value unit.

## Discussion

In this quality improvement study of patients seeking elective hernia repair, a preoperative optimization clinic was able to actively engage 165 high-risk patients, 15 of whom were subsequently eligible to undergo surgical hernia repair after health optimization. Over the course of 12 months, the rate of emergency surgery in this cohort was 3%, and surgical yield in the standard hernia clinic was increased by 19%. These data suggest that delaying surgery for the supported preoperative optimization of high-risk patients with hernias is a safe and financially viable strategy to scale across institutions to enable surgeon-led risk mitigation for patients before surgery.

An emergency surgery rate of 3% was documented in this patient cohort, which is at or below previously published rates of emergency surgery in patients undergoing nonoperative management.^14,18,19^ This low rate of emergency surgery may be associated with the longitudinal follow-up that increased patient access to clinicians and surgical teams in the event of worsening health status. The utility of nonoperative management has gained traction as various studies have minimized concerns that delaying surgery will affect patient quality of life. Among high-risk surgical patients who require optimization, a temporary surgical delay in favor of nonoperative management has been reported to not decrease satisfaction or function.^20^ Nevertheless, our study demonstrates that a small proportion of patients may be adversely affected while deferring surgery. In our clinic, the potential for this risk was mitigated by the advanced practice clinician being well trained in hernia management and acute hernia emergencies. In addition, there was always a surgeon available for immediate consultation or questions during the clinic. Patients seen by the physician assistant also had the opportunity to be presented at a complex hernia case conference if it was thought that they could benefit from the input of a multidisciplinary group of clinicians. However, these results indicate that closer follow-up during this period may be warranted to intervene preemptively for patients who have a major change in symptoms. Even if a patient has not completely quit smoking or lost a substantial amount of weight, early intervention in the setting of worsening or debilitating symptoms may prevent a surgical emergency and, therefore, may still result in a better outcome.

The number of patients successfully crossed over to surgery during this study period supports the known effectiveness of preoperative optimization on health outcomes.^15^ However, these results also highlight the difficulty of changing health behaviors. Some may see a 9.1% crossover rate from nonoperative to operative status as dismal results; however, this clinic was designed to capture nonoperative candidates, because of the presence of relative contraindications that could undergo mitigation. We view this intervention and the subsequent health changes exhibited as an improvement in management compared with patients being seen in a surgery clinic where they would have been declined surgery and likely provided no further support in their health goals. The support provided through our clinic, to patients who may not be otherwise engaged in quality care, may also be the reason that patient retention was high, even when they were not offered surgery immediately. One year after clinic implementation only 1 patient underwent elective surgery at a different institution, which is lower than previously reported rates of seeking surgical care elsewhere during nonoperative management.^21^ Fundamental lifestyle changes are challenging, with low success in the general population. Therefore, it is important to note that this program was able to leverage interactions in the preoperative period to provide an intervention to a patient population that may not have taken steps otherwise. In the US, despite the majority of cigarette smokers reporting a desire to quit, only 7% of smokers successfully quit each year.^22^ With the built-in support through our optimization program and institutional resources, almost 14% of enrolled active smokers were able to successfully quit smoking in an early-phase, small-scale pilot initiative. Having a tangible goal—namely, undergoing surgery—likely contributed to the high rate of cessation documented. This aligns with the goals of the optimization clinic, taking advantage of the opportunity that the preoperative period represents. A surgical episode often serves as a teachable moment, and it has previously been shown that patients undergoing surgery are more likely to achieve important health behavior improvements compared with nonoperative control patients.^7,8^ In addition, self-motivated weight loss is incredibly difficult to achieve without support. Even in structured weight loss programs, few patients are able to achieve sustained weight reduction, with patients reporting only a 3% to 6% weight reduction in general.^23^ Our intervention provided a framework of exercise and nutrition goals but was also able to provide eligible participants the opportunity to enroll in a bariatric surgery program. For individuals with obesity, bariatric surgery is consistently the most effective weight loss strategy, with additional known reduction in comorbidities and all-cause mortality.^24,25^ In the US, bariatric surgery is underused as a weight loss resource, with only approximately 1% of eligible patients undergoing surgery annually.^26^ In our cohort, 4.7% of individuals with obesity in the optimization program became eligible for surgery through successful weight loss, and 10.8% became actively enrolled in a bariatric surgery program. Therefore, within the larger context of the difficulty achieving successful lifestyle change, these modest results represent a substantial success after only a simple engagement through the medical system.

Despite these achievements, there is a substantial opportunity to improve the outcomes of this pilot-scale optimization clinic and maximize an interaction with a patient population that may be willing to change. The scope of this pilot initiative was designed to be narrow, focusing on weight, tobacco use, and age-related frailty to allow easy implementation, and to test a model of the management of high-risk patients requesting elective surgery. As such, the inclusion criteria outlined in this study are not meant to be prescriptive but rather to provide a framework for how to manage high-risk patients that can be adapted to fit the processes of other institutions. For a high-throughput institution, age 75 years or older was chosen as clinic inclusion criterion, but for other institutions it may be advantageous to include different or a broadened scope of risk factors according to their capacity. Expanded clinic inclusion criteria could allow a more nuanced approach to the screening and optimization of frailty as a surgical risk factor, beyond the current approach of simply using age 75 years or older as a screening criterion. In addition, expanding the focus to other easily intervenable chronic conditions, such as uncontrolled diabetes, untreated hypertension, pulmonary deconditioning, and even medication management such as steroid use, has the potential to greatly expand the impact of the clinic and may be appropriate to implement at other institutions. Alternatively, a more selective approach may be more feasible in resource-limited settings and, ultimately, may be more successful. For example, patients could be screened for their readiness to quit tobacco before dedicating time and resources to intervention. Although diabetes was not used as a qualifying diagnosis for inclusion because of the pilot nature of the clinic and workflow limitations, patients who presented with a comorbid diagnosis of uncontrolled diabetes were provided with resources such as nutrition advice, dietician referrals, and assistance in blood glucose management. However, institutions with different workflows for triaging patients may wish to alter the qualifying inclusion criteria to best fit their system. For example, there is an abundance of literature suggesting that hernia repair should be deferred for patients with a hemoglobin A_1c_ greater than 8.0%; therefore, this could reasonably be used as a screening target to enroll patients and address uncontrolled diabetes.^27^ To further capitalize on the known patient acceptability to change during the preoperative period, clinicians staffing the clinic can be further trained in motivational interviewing and other strategies on how to best leverage the opportunity in these patient encounters. In addition, this pilot intervention provided the opportunity to trial the specificity and sensitivity of a new triage algorithm and workflow adjustment in the call center. This simple intervention, a binary screening for the presence of 3 risk factors, was effective in capturing patients with both clinical hernias and high-risk characteristics. However, inherent limitations of patient-reported data and screening by nonmedical professional staff resulted in 6 patients with groin hernias presenting to the optimization clinic, although the program was designed for patients with abdominal hernias. Although this represents a small number of patients, and likely negligible impact on the clinic, it provided valuable feedback for additional details to ascertain in the screening process, such as more nuanced hernia location and potentially incorporating a review of the medical record for any prior classification of the hernia. In addition, this pilot clinic was not designed to capture every patient with high-risk characteristics, because it was trialed as a half-day clinic with limited capacity for the first year. All patients had the choice to either see a surgeon, with the knowledge that they had a relative contraindication to surgery, or to be seen in the optimization clinic before surgical evaluation. Every patient who requested an appointment received one. Because of limitations of the call center, we were not able to track the number of patients who declined to enroll in the optimization program, although this is a future plan in the expansion of this work and the evaluation of longer-term implementation outcomes. However, the increase in operative yield demonstrates that a substantial number of noneligible surgical candidates were rerouted from the surgical clinic.

During the implementation period of the preoperative optimization clinic, surgical yield in the standard hernia clinic increased, perhaps because high-risk patients who were unlikely to be operative candidates were preemptively routed to the optimization clinic. There was a 58% increase in RVUs attributed to hernia operations. Surgical prehabilitation has previously been shown to reduce costs at the patient level^28^; the current findings demonstrate that prehabilitation is also associated with financial gain at the institutional level or, at the very least, that such a program is not cost-prohibitive. The optimization outcomes presented here were achieved through a half-day clinic, through which the cost of clinician salary (0.1 full-time equivalent or approximately $10 000) and clinic space were offset by financial gain for the institution. The financial gains were attributed solely to the optimization clinic intervention, as there were no other changes to the division or competing efforts in this domain at the institution or state level during this period. Institutional costs may have the potential to be further decreased with the use of a telehealth model. These results demonstrate that a preoperative optimization clinic is a financially worthwhile and sustainable investment for the surgical division. The main requirement for implementation is an institutional workflow adjustment in which a triage algorithm is applied to patients requesting elective surgery. Almost any system type could implement this framework, within the constructs of institution-specific workflow constraints. For example, this investment could be a shared resource across multiple surgical specialties that may have similar preoperative optimization needs.

Nearly one-tenth of patients who attended this financially viable model clinic went on to undergo surgical hernia repair, and further adoption of this approach could maximize patient flow in constrained systems without negatively affecting financial interests. This potential for mutual benefit may allow the focus of patient optimization to broaden from simply changing patient behaviors to also improving surgeon decision-making regarding the performance of elective operations on high-risk patients.

Up to one-quarter of patients undergoing hernia repair have critical risk factors at the time of surgery.^3^ This variability in clinical decision-making is likely associated with numerous barriers, such as clinical and financial resources required to optimize patients, as well as fears that compensation and future referrals may be lost by not offering a patient an operation up front. Optimizing patients for surgery is a time-intensive process, but our findings suggest that the resources required are offset by increased economic gain from surgical yield. Moreover, delaying surgery was not associated with negative health outcomes compared with the known preoperative complication rates of patients with hernias at large. By incentivizing a focus on ensuring that patients are optimally prepared for surgery, institutions could reduce their own back-end costs while simultaneously mitigating surgeon concern that optimization would result in lost business. Efforts aimed at improving and even standardizing decision-making with regard to preoperative optimization may represent one of the largest levers by which to improve overall surgical quality. The implementation of a preoperative optimization clinic has the potential to maximize patient flow in a constrained system by sustainably increasing patient capacity supported by net revenue gained.

### Limitations

This study is not without limitations. This cohort included patients with a variety of hernia types, recurrences, and severity, including ventral, incisional, and groin hernias, which may limit the generalizability of results regarding risk of delayed surgery. Moreover, the benefit derived from preoperative optimization may be substantially different between patients with relatively straightforward, small ventral hernias and those with complex, recurrent ventral hernias. Because the benefit of optimization extends beyond simply improved surgical outcomes and has the potential to also improve a patient’s overall health and longevity, all patients regardless of hernia characteristics were included in this study. Clinic implementation was also a test of the effectiveness of one simple workflow adjustment in the call center triage. This initial screening was conducted through a call center by persons without a medical degree who were responsible for fielding large volumes of calls from patients across multiple specialties each day. This limited the ability to obtain the information required to determine the hernia risk level or differentiate the severity of additional comorbidities. Achieving this level of clinical nuance through the current call center staff and workflow would have required further institutional investment, as this level of detail obtainment is not currently feasible through a call center staffed by nonmedical professionals. In settings where resources may be more limited or the triage workflow is different, it may be appropriate to change these criteria or even limit inclusion to only those more complex patients who may derive the most benefit from optimization. In addition, there are other avenues to reduce surgical risk for patients with comorbid conditions besides preoperative risk mitigation. Pursuing less invasive hernia repair, for example under local anesthesia or with conscious sedation, provides another pathway for these patients to become surgical candidates. An additional limitation of our study is the 1-year follow-up time, which may have failed to capture the full trajectory of all patients. However, this pilot study was designed to address the safety profile of this intervention and whether a surgical delay would increase risk of hernia incarceration or emergency surgery, which is a common concern cited by surgeons in response to interventions of this type. In an effort to address safety of a preoperative optimization clinic before further scaling of the intervention, we designed this study to report on 1-year outcomes. We believed that 1 year of follow-up was sufficient because delaying or foregoing hernia surgery in high-risk patient populations has previously been found not to be associated with increased risk.^14,29^ The follow-up time also limits our knowledge of the long-term trajectory of patients who quit smoking to undergo surgery, specifically, an understanding of whether they relapsed after their surgery was performed. Furthermore, although our small patient cohort is a notable limitation, it was representative of the local geographical population, and patients who left the state of Michigan for care would not be captured in the study population.

## Conclusions

In this study, nearly 10% of patients with hernias and high-risk health characteristics who attended a pilot preoperative optimization clinic went on to undergo elective surgical repair of their hernias, and 3% required emergency surgery The low rate of unintended health consequences during clinic enrollment suggests that surgery can safely be delayed to mitigate risk factors and improve care for high-risk surgical patients. The increased RVU generation suggests that the preoperative optimization clinic substituted patients who would not be eligible for surgery from the standard hernia clinic with those more likely to undergo surgery. This suggests that future efforts toward improving decision-making in regard to preoperative optimization may represent one of the largest levers by which to improve overall surgical quality. Further work is needed to understand how to increase the success of optimization for high-risk patients.

## Notes

Supplement. **eTable.** Hospital Encounters During Optimization Clinic Enrollment Period
