# Supplementary material for: Outcomes of a Presurgical Optimization Program for Elective Hernia Repairs Among High-risk Patients
Source: JAMA Netw Open. 2021 Nov 1;4(11):e2130016. doi: 10.1001/jamanetworkopen.2021.30016 (PMC8561332; doi:10.1001/jamanetworkopen.2021.30016)
Supplement: Supplement. — eTable. Hospital Encounters During Optimization Clinic Enrollment Period [file jamanetwopen-e2130016-s001.pdf]

Supplemental Online Content

Delaney LD, Howard R, Palazzolo K, et al. Outcomes of a presurgical optimization program for elective hernia repairs among high-risk patients. *JAMA Netw Open*. 2021;4(11):e2130016. doi:10.1001/jamanetworkopen.2021.30016

eTable. Hospital Encounters During Optimization Clinic Enrollment Period

This supplemental material has been provided by the authors to give readers additional information about their work.

© 2021 Delaney LD et al. *JAMA Network Open.*

eTable. Hospital Encounters During Optimization Clinic Enrollment Period

All Cause Hospital Admissions	22 (13.3)
Non-Hernia ED Presentations [a]	30 (18.2)
Hernia ED Presentations [b]	11 (6.7)
Hernia Incarceration Event	5 (3)
Emergency Surgery	5 (3)

[a] 'Non-Hernia ED Presentations' refers to any presentation to an emergency department during the follow-up time that was not due to patient concern for hernia pain or a hernia-related complication.
[b] 'Hernia ED Presentations' refers to any patient presentation to an emergency department during the follow-up time that was due to to patient concern for hernia-related pain or complication.

© 2021 Delaney LD et al. *JAMA Network Open.*
